# Abdominal skin subcutaneous fat thickness over the gestational period in Korean pregnant women: a descriptive observational study

**DOI:** 10.4069/kjwhn.2021.12.12

**Published:** 2021-12-29

**Authors:** Moon Sook Hwang

**Affiliations:** College of Nursing, Woosuk University, Wanju, Korea

**Keywords:** Abdomen, Pregnancy, Skinfold thickness, Subcutaneous injection, Ultrasonography

## Abstract

**Purpose:**

Although insulin is usually injected into the abdominal subcutaneous fat, in pregnancy women tend to avoid abdominal injections due to concern about fetal damage. Prior studies have been limited to only measuring skin-subcutaneous fat thickness (S-ScFT) at one site at specific pregnancy points. This study aimed to measure S-ScFT across several abdominal sites and over the gestational period in Korean pregnant women. This can identify which site would be relatively safe for subcutaneous injection during pregnancy.

**Methods:**

Healthy women over 24 weeks of pregnancy in Korea were invited to voluntarily participate in this descriptive study. For the 142 women, S-ScFT of 12 sites in the abdomen were measured by ultrasound, several times over the pregnancy. Each incidence was treated as a case and a total of 262 cases were analyzed.

**Results:**

The mean S-ScFT during pregnancy was 1.14±0.47 cm (1.25±0.54 cm at 24^+0^–27^+6^ weeks; 1.17±0.48 cm at 28^+0^–31^+6^ weeks; 1.09^+0^.40 cm at 32^+0^–35^+6^ weeks; and 1.06±0.47 cm at 36^+0^–40 weeks of pregnancy). Most S-ScFT were thicker than 10 mm. But S-ScFTs in the lateral abdomen and some sites were suboptimal (<6 mm), especially in the pre-pregnancy underweight body mass index group, who had a high rate of suboptimal thickness (27.1% overall and 33.9% in the lateral side).

**Conclusion:**

The whole abdomen seems to be appropriate for subcutaneous injection in most Korean women during pregnancy, with a 4 to 5-mm short needle. However, for the lateral abdomen, making the skin fold might be needed for fetal safety.

## Introduction

The prevalence of gestational diabetes mellitus (GDM) has been increasing, which is parallel to the increase of advanced maternal age pregnancy [1]. Proper control of blood glucose level for GDM patients is important for the health of the mother and fetus, and insulin therapy is preferred to oral hypoglycemic agents for treating hyperglycemia in terms of safety [1,2].

Insulin is usually injected into the abdominal subcutaneous fat, comparing with thigh and upper arm, not only because it is convenient but also this site is where insulin can be absorbed the most uniformly [3]. Since short injection needles (4–5 mm) were developed recently, injecting insulin on the side abdomen has been recommended for pregnant women with GDM [4,5].

However, most GDM patients inject insulin on their upper arm or thigh due to anxiety of hurting their fetus with the needle [3]. Since the abdominal skin-subcutaneous fat thickness (S-ScFT) of the pregnant women becomes thinner as the fetus grows [6], the needle can pass through the subcutaneous fat and reach the muscle layer where many blood vessels are distributed. Insulin quickly absorbed into the muscle layer may cause hypoglycemia [7,8], and rotational insulin injection is recommended to prevent lipohypertrophy [9]. Thus, care is required when insulin injections are needed in GDM.

S-ScFT has been reported as slightly thicker in GDM than in normal pregnant women when measured in early pregnancy [10] and at around 24 weeks [11]. However, most prior research on GDM has only used measurement from a single site, the skin at the rectus ab¬dominis muscle at 1 cm above the umbilicus [10,12]. Other studies on pregnant women have also been limited to other single sites, the midline on the upper abdomen [13] and mid‐sagittal and superior to the symphysis pubis, measuring in the midline through the linea alba [11]. Furthermore, despite a few studies that checked abdominal S-ScFT according to gestational weeks [6,13], to our knowledge, there have been no such studies in Korea with GDM or pregnant women.

Therefore, this research aimed to identify S-ScFT across several abdominal sites and through several measurements over the gestational period (GP) in Korean pregnant women. As participation of women with GDM would not be feasible and their S-ScFT could be considered slightly thicker than normal pregnant women, we chose normal pregnant women as a conservative sample. Ultimately, this study could identify which abdominal site would be relatively safe for Korean women when needing subcutaneous injection during pregnancy.

## Methods

Ethics statement: This research was approved by the Institutional Review Board of Woosuk University (No. 2020-12). Informed consent was obtained from all participants.

### Study design

This descriptive study reports the possible subcutaneous injecting site on measurement of S-ScFT of 12 sites in the abdomen during pregnancy.

### Participants

The data was collected in the Hanbyul Women’s Hospital located in Jeonju, Korea from September 1, 2020 to February 20, 2021. The research enrolled 142 normal pregnant women over 24 weeks’ pregnancy. As women were measured several times over pregnancy, each incidence was treated as a case. A total of 262 cases measuring the S-ScFT of 12 abdominal sites and were used for analysis.

Inclusion criteria were as follows: (1) over 24 weeks’ pregnancy, according to screening recommendations for GDM at 24 to 28 weeks of pregnancy [14]; (2) married pregnant women aged 19 years and over; (3) cephalic fetal presentation, normal range cardiac sound, and with no problem in fetal movement; and (4) over 50% fetal weight for GP by sonography.

Exclusion criteria were pregnant women (1) in the high-risk group, in terms of multiple fetus indicators; (2) with opened cervix or amniotic membrane rupture; or (3) with warts, bruises, pierced wounds, and scratches on the abdomen.

Estimating the effect size of 0.25, significance level of 0.05, power of 0.90, and group number of four using the G*Power 3.1.9.7 program to ensure a suitable sample size for the analysis of variance (ANOVA) method to examine the difference of the S-ScFT by the body mass index (BMI), a minimum of 232 cases were required. Consequently, 262 cases from the research 142 participants were sufficient for analysis.

### Study setting and measurements

#### Grouping by gestational period

As most pregnant women get their diabetes screening tests in the 24th week of pregnancy and come to a regular follow-up after 4 weeks, we divided the GP from 24 to 40 weeks into four groups, that is 24 to 27^+6^ weeks, 28 to 31^+6^ weeks, 32 to 35^+6^ weeks, and 36 to 40 weeks.

#### Pre-pregnancy body mass index

Biophysically BMI is the method to assume the subcutaneous fat thickness reliably. Participants were divided into BMI four groups based on their pre-pregnancy weight [15]: underweight, <18.5 kg/m2; normal weight, 18.5 - 22.9 kg/m2; overweight, 23 – 24.9 kg/m2; and obese, ≥25 kg/m2.

#### Skin-subcutaneous fat thickness

**Figure 1.**
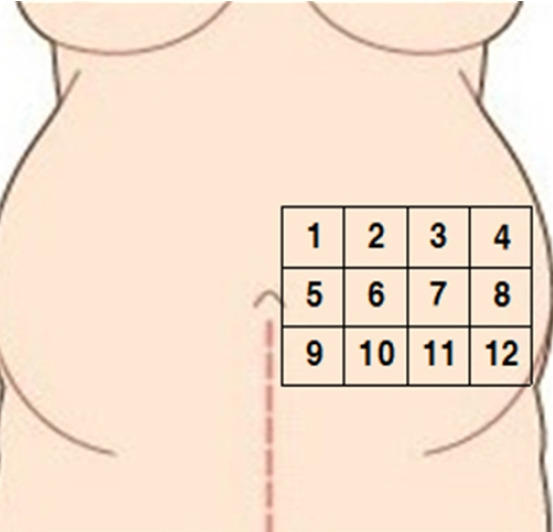
Measurement sites of skin-subcutaneous fat thickness on the abdomen.

The S-ScFT in this research refers to the value summing the skin thickness and subcutaneous fat thickness. We selected and measured the left side of umbilicus line because fetal position and presentation are more commonly left occipital than right occipital side [16]. The left side abdomen was divided into three rows and four columns, totaling 12 sections. The upper boundary was the lower margin of rib and the lower boundary was the upper margin of iliac crest. From the first row, each section was numbered from medial to lateral (Figure 1).

**Figure 2.**
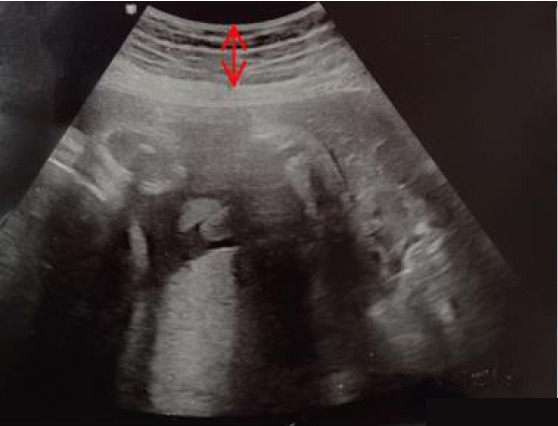
Axial ultrasound image. Arrow indicates skin-subcutaneous fat thickness.

The S-ScFT was measured using ultrasonic equipment (HS60, Samsung Medison, Seoul, Korea) (Figure 2), and the results are described in centimeters across 12 sites on the abdomen. A total of five skilled obstetricians performed the exam. In order to enhance the reliability among measurers, we guided the representative doctor to select the median values out of the three measurements per every 12 measuring area. For accurate measurements, not only examiners put in the effort not to press the abdominal skin but also the participant was instructed to stop breathing for a moment. A basic frequency was set to 10 MHz but changing the frequency within the range of 7 to 12 MHz was possible to acquire a clear and precise ultrasonic image. The other status, including fetal size, was checked and recorded as well.

Insulin should be injected ScFT through the skin and recently the shortest insulin needle length developed is 4 mm. On the other hand, skin thickness is 2 mm regardless of other variables [16,17]. Also, distance considering connection part for between pillar and hub of needle also, pressure on the skin during injection is 2 mm [18]. So, the judgment criteria for S-ScFT were decided as 6 mm over in this research, and <6 mm was categorized as suboptimal skin-subcutaneous fat thickness.

We also assessed general characteristics (age, residence, health insurance, and family history of diabetes mellitus) by interview and fetal growth curve by medical record after receiving consent.

### Statistical analysis

Using the IBM SPSS ver. 25 (IBM Corp., Armonk, NY, USA), categorical variables were presented as frequency and percentage, and continuous variables were presented as mean±standard deviation. The differences in S-ScFT according to the general and obstetrical characteristics were analyzed by t-test or ANOVA. Post-hoc tests were conducted for three and more variables using the Scheffé test.

## Results

### Difference of skin-subcutaneous fat thickness depending on general characteristics

As shown in Table 1, the mean age of the 142 participants was 32.14±4.11 years, and roughly one-fifth (19.0%) were over 35 years. Women of low socioeconomic status had thicker S-ScFT than normal women (1.49±0.04 cm vs. 1.21±0.50 cm, *p*<.001)

According to pre-pregnancy weight classification, the S-ScFTs of obese and overweight groups were thicker than those of women who were normal and underweight (obese, 1.53±0.53 cm; overweight, 1.37±0.51 cm; normal, 1.09±0.41 cm; and underweight, 0.80±0.42 cm; *p*<.001) (Table 1).

### Skin-subcutaneous fat thickness of each abdominal site according to gestational periods

Fifty-seven cases out of 262 cases accounted for 24^+0^ to 27^+6^ GP, 69 cases for 28^+0^ to 31^+6^ GP, 67 cases for 32^+0^ to 35^+6^ GP, and 69 cases for 36^+0^ to 40 GP. The S-ScFT showed tendency to decrease as GPs increased (24 to <28 weeks, 1.25±0.54 cm; 28 to <32 weeks, 1.17±0.48 cm; 32 to <36 weeks, 1.09±0.40 cm; and 36 to 40 weeks, 1.06±0.47 cm). The mean S-ScFT of site 6 (medial abdomen) was the thickest (1.30±0.56 cm) and that of site 12 (nearest to the anterior superior iliac spine; the most lateral site) was the thinnest (0.93±0.46 cm) (Table 2).

### Incidence of skin-subcutaneous fat thickness below 6 mm

The incidence of suboptimal S-ScFT (<6 mm) was 14.9%. First, we analyzed S-ScFT according to pre-pregnancy BMI. The incidence of suboptimal S-ScFT was higher in women who had been of underweight (27.1%) compared to the overweight group (7.9%) and the obese group (6.7%). And normal weight group also showed 18.1% of suboptimal S-ScFT. The frequency of suboptimal S-ScFT was particularly high on site 12 in 53.6% of the underweight group.

Next, upon analysis of S-ScFT according to GP, the frequency of S-ScFT below 6 mm increased slightly from early 10% until 36 weeks of GP but increased sharply over 36 weeks to 20.5%. All lateral parts, including sites 3, 4, 7, 8, 11, and 12, showed more than 20% of the incidence of suboptimal S-ScFT over 36 weeks. In both BMI and GP classification, the suboptimal S-ScFT was more frequent in these lateral (outer) side than in the medial (inner) side of the abdomen (Table 3).

## Discussion

In this study, the S-ScFT generally decreased as the pregnancy progressed. This was in line with Selovic et al.’s report [13] that S-ScFT did not show any difference until 20 weeks of pregnancy, but became thinner after that. By 20 weeks of pregnancy, the uterus rests against the lower portion of the front of the abdominal wall, causing it to bulge forward noticeably. Due to the tension applied to the abdomen according to fetal growth, the ScFT of the abdomen becomes thinner. In the same aspect, fetal estimated body weight appears to have presented a negative correlation with S-ScFT.

In this research, S-ScFT of the pre-pregnancy underweight and normal BMI groups were significantly thinner than that of the pre-pregnancy overweight and obese BMI groups. As is generally known, the amount of subcutaneous fat is proportional to BMI [19-23]. Both domestic research [10,12] and international research [6,11,13] targeting pregnant women revealed similar results. On the other hand, Kennedy et al. [6] reported that S-ScFTs of the overweight and obese group became thinner with GP going by, while S-ScFT of the normal group kept stable. As such, the results according to researchers were different. This study showed a tendency that high pre-pregnancy BMI was associated with thick S-ScFT and decreased as GP increased. Considering that S-ScFT became thinner than 6 mm in late pregnancy (20.5% in 36–40 weeks of GP), special attention is needed at the time of subcutaneous injection considering the proportion of suboptimal S-ScFT after 36 weeks.

In this study, the average S-ScFT was more than 1 cm during the overall pregnancy period, which suggests it is theoretically possible to do subcutaneous injections safely with 4 to 6-mm needles. The frequency of suboptimal S-ScFT was higher in the pre-pregnancy underweight group (27.1%) than in the overall sample (14.9%), which is similar to the pattern reported for women with type 2 diabetes, 35.3% in the underweight BMI group and 12.8% in the overall sample [3]. As suboptimal S-ScFT was high in the outer side (lateral abdomen), especially 33.9% in the underweight group, insulin injected into this area may potentially involve the muscular layer’s abundant vessels, which can lead to absorption of insulin too rapidly [5]. As such, in underweight cases, it seems to be safer to inject insulin into upper arm or thigh than abdomen, but self-injections on the upper arm or thigh can be inconvenient. Although the growing abdomen bulges forward as pregnancy progresses, the lateral side is relatively loose, as evidenced by less suboptimal S-ScFTs on the lateral side compared to the central side of the abdomen. This means that subcutaneous injections to pregnant women’s lateral abdomen (sites 3, 7, 8, 11) making a skinfold by pinch up during pregnancy is possible for all BMI groups, and more so necessary for underweight women. However, sites 4 and 12 are not recommended, considering they are not easy to access for self-injections and tended to have suboptimal thickness in this study.

Economic status was also a statistically significant differing factor for S-ScFT in this study. Study findings that S-ScFT of economically vulnerable cases were thicker than those of having general insurance type, is consistent with prior research in Korea of type 2 diabetes [3] and with Korean pregnant women [24]. The high correlation between pre-pregnancy BMI and S-ScFT is also related to this. Overweight and obese women tend to experience prolonged delivery time compared with normal-weight women [25]. Therefore, support for managing BMI before getting pregnant is important.

There were some limitations of the study. It was not checked whether abdominal circumference increased or decreased according to the amniotic fluid amount, nor was fetal presentation, which could potentially affect S-ScFT. Also, only the left side of the umbilicus line was measured. Although normal pregnant women as a conservative sample were studied, in reality women with GDM who require insulin injection may have less proportion of suboptimal thickness. As such, interpretation and generalization are limited. Despite these limitations, this study provides data on overall abdominal sites across progression of pregnancy in Korean women.

In conclusion, the S-ScFT for 12 abdominal sites were measured with ultrasound to see if a safe subcutaneous abdominal injection is possible in pregnancy. This study found the mean S-ScFT was over 1 cm from 24 weeks to 40 weeks and insulin injections using a 4- to 6-mm needle could be possible in general cases. However, there were cases in which S-ScFT became thinner than 6 mm, i.e., in late pregnancy (20.5% in 36–40 weeks of GP) and in the pre-pregnancy underweight group (27.1%). Suboptimal thickness was also more frequent in the lateral abdomen. For the safety of the pregnant women and fetus, injecting with two-fold of skin on the lateral side of abdomen (sites 3, 7, 8, 11) can be recommended.

## Figures and Tables

**Table 1.** Difference of skin-subcutaneous fat thickness by general characteristics of participants (N=142)

Variable	Categories	n (%)	Mean±SD	t/F (*p*)
Age (year)	<5	115 (81.0)	1.22±0.51	0.36 (.359)
	≥35	27 (19.0)	1.18±0.473	
			32.14±4.11	
Residential area	City	98 (69.0)	1.22±0.52	0.45 (.327)
	Rural	44 (31.0)	1.18±0.48	
Health insurance	General (general)	140 (98.6)	1.21±0.50	5.37 (<.001)
	Medicaid (poor)	2 (1.4)	1.49±0.04	
Pre-pregnancy BMI (kg/m^2^)^†^	Underweight^a^	15 (10.6)	0.80±0.42	9.57 (<.001)
	Normal weight^b^	70 (49.3)	1.09±0.41	a < b,c < d
	Overweight^c^	28 (19.7)	1.37±0.51	
	Obesity^d^	29 (20.4)	1.53±0.53	
			22.54 ± 3.61	
Familial diabetes mellitus	No	119 (83.8)	1.20±0.51	0.57 (.284)
	Yes	23 (16.2)	1.27±0.48	
Menarche age^‡^ (year)	<16	126 (88.7)	1.21±0.51	0.26 (.397)
	≥16	16 (11.3)	1.24±0.45	
			14.06±1.47	
Regularity of menstruation^‡^	Yes	112 (79.4)	1.20±0.50	0.66 (.255)
	No	29 (20.6)	1.27±0.50	
Present pregnancy	Primipara	98 (69.5)	1.21±0.48	0.14 (.443)
	Multipara	43 (30.5)	1.22±0.56	
Ratio of fetal growth^‡^	AGA (50-89%)	124 (89.2)	1.24±0.50	1.55 (.061)
	LGA (≥90%)	15 (10.8)	1.03±0.43	

AGA: Appropriated for gestational age; BMI: body mass index; LGA: large for gestational age.

† BMI: underweight, <18.5 kg/m^2^; normal weight, 18.5 -22.9 kg/m^2^; overweight, 23-24.9 kg/m^2^; obesity, ≥25 kg/m^2^.

‡ Missing data were excluded.

**Table 2.** Suboptimal skin-subcutaneous fat thickness on each abdominal site by gestational period for all cases (N=262)

Division	24^+0^–27^+6^ weeks (n=57)		28^+0^–31^+6^ weeks (n=69)		32^+0^–35^+6^ weeks (n=67)		36^+0^–40 weeks (n=69)		Total	
	Mean±SD	Range	Mean±SD	Range	Mean±SD	Range	Mean±SD	Range	Mean±SD	Range
Site 1	1.32±0.58	0.45–2.73	1..21±0.52	0.31–2.49	1.05±0.39	0.26–2.17	0.98±0.42	0.28–2.35	1.13±0.49	0.26–2.73
Site 2	1.39±0.58	0.50–3.11	1.30±0.52	0.40–2.56	1.17±0.48	0.26–2.34	1.09±0.46	0.36–2.32	1.23±0.52	0.26–3.11
Site 3	1.30±0.59	0.38–2.69	1.24±0.55	0.33–2.94	1.12±0.49	0.34–2.42	1.04±0.48	0.36–2.38	1.17±0.53	0.33–2.94
Site 4	1.10±0.52	0.33–2.26	1.12±0.50	0.30–2.43	0.98±0.46	0.28–2.94	0.88±0.39	0.31–2.27	1.02±0.48	0.28–2.94
Site 5	1.43±0.60	0.37–3.18	1.30±0.55	0.33–2.40	1.23±0.45	0.21–2.31	1.16±0.57	0.24–2.84	1.27±0.55	0.21–3.18
Site 6	1.41±0.66	0.33–3.09	1.31±0.53	0.38–2.65	1.29±0.47	0.37–2.40	1.22±0.57	0.26–2.62	1.30±0.56	0.26–3.09
Site 7	1.29±0.59	0.45–2.71	1.18±0.54	0.26–2.51	1.10±0.44	0.45–2.08	1.10±0.53	0.31–2.38	1.16±0.53	0.26–2.71
Site 8	1.17±0.56	0.28–2.61	1.13±0.49	0.32–2.47	1.02±0.44	0.31–2.08	0.99±0.47	0.29–2.23	1.07±0.49	0.28–2.61
Site 9	1.21±0.64	0.25–2.87	1.14±0.49	0.40–2.20	1.14±0.42	0.39–2.15	1.18±0.56	0.26–2.65	1.16±0.53	0.25–2.87
Site 10	1.13±0.62	0.06–2.87	1.11±0.49	0.33–2.15	1.08±0.44	0.26–2.13	1.13±0.57	0.26–2.61	1.11±0.53	0.06–2.87
Site 11	1.12±0.56	0.21–2.58	1.09±0.50	0.33–2.18	1.02±0.42	0.33–2.14	1.06±0.56	0.21–2.77	1.07±0.51	0.21–2.77
Site 12	0.95±0.50	0.18–2.56	0.94±0.46	0.12–2.11	0.90±0.43	0.07–1.98	0.92±0.47	0.26–2.14	0.93±0.46	0.07–2.56
Total	1.25±0.54	0.41–2.59	1.17±0.48	0.41–2.27	1.09±0.40	0.37–1.96	1.06±0.47	0.32–2.38	1.14±0.47	0.32–2.59

**Table 3.** Incidence of suboptimal skin-subcutaneous fat thickness on abdominal site by body mass index and gestational period for all cases (N=262)

Division	Body mass index^†^					Gestational period (week)			
	Underweight (n=28)	Normal (n=133)	Overweight (n=54)	Obesity (n=47)	24^+0^–27^+6^ (n=57)	28^+0^–31^+6^ (n=69)	32^+0^–35^+6^ (n=67)	36^+0^–40 (n=69)	Total
Site 1	6 (21.4)	20 (15.0)	5 (9.3)	3 (6.4)	5 (8.8)	9 (13.0)	7 (10.4)	13 (18.8)	34 (13.0)
Site 2	3 (10.7)	16 (12.0)	4 (7.4)	5 (10.6)	3 (5.3)	5 (7.2)	8 (11.9)	12 (17.4)	28 (10.7)
Site 3	5 (17.9)	22 (16.5)	3 (5.6)	4 (8.5)	3 (5.3)	7 (10.1)	10 (14.9)	14 (20.3)	34 (13.0)
Site 4	12 (42.9)	26 (19.5)	4 (7.4)	4 (8.5)	7 (12.3)	9 (13.0)	11 (16.4)	19 (27.5)	46 (17.6)
Site 5	6 (21.4)	12 (9.0)	5 (9.3)	1 (2.1)	3 (5.3)	5 (7.2)	4 (6.0)	12 (17.4)	24 (9.2)
Site 6	2 (7.1)	15 (11.3)	3 (5.6)	1 (2.1)	4 (7.0)	4 (5.8)	4 (6.0)	9 (13.0)	21 (8.0)
Site 7	7 (25.0)	25 (18.8)	3 (5.6)	4 (8.5)	6 (10.5)	9 (13.0)	10 (14.9)	14 (20.3)	39 (14.9)
Site 8	9 (32.1)	28 (21.1)	4 (7.4)	3 (6.4)	8 (14.0)	9 (13.0)	12 (17.9)	15 (21.7)	44 (16.8)
Site 9	10 (35.7)	25 (18.8)	6 (11.1)	3 (6.4)	12 (21.1)	10 (14.5)	10 (14.9)	12 (17.4)	44 (16.8)
Site 10	7 (25.0)	28 (21.1)	4 (7.4)	2 (4.3)	11 (19.6)	9 (13.0)	7 (10.4)	14 (20.3)	41 (15.6)
Site 11	9 (32.1)	31 (23.3)	6 (11.1)	3 (6.4)	11 (19.3)	10 (14.5)	12 (17.9)	16 (23.2)	49 (18.7)
Site 12	15 (53.6)	39 (29.3)	6 (11.1)	4 (8.5)	11 (19.3)	17 (24.6)	16 (23.9)	20 (29.0)	64 (24.4)
Total	91 (27.1)	287 (18.1)	53 (7.9)	37(6.7)	84 (12.3)	103 (12.4)	111 (13.8)	170 (20.5)	468 (14.9)
Central area	34 (20.2)	116 (14.5)	27 (8.0)	15 (6.9)	37 (10.8)	42 (10.1)	40 (10.0)	72 (17.4)	192 (12.2)
Lateral area	57 (33.9)	171 (21.4)	26 (7.7)	22 (10.2)	46 (13.5)	61 (14.7)	71 (17.7)	98 (23.7)	276 (17.6)

† BMI: underweight, <18.5 kg/m^2^; normal weight, 18.5 – 22.9 kg/m^2^; overweight, 23-24.9 kg/m^2^; obesity, ≥25 kg/m^2^.
